# Interstitial Lung Disease in the United States: CDC Mortality Trends (1999–2024)

**DOI:** 10.3390/arm94040051

**Published:** 2026-07-24

**Authors:** Palak Grover, Rahul Jain, Gurleen Kaur, Bipneet Singh

**Affiliations:** 1 Department of Internal Medicine, Henry Ford Jackson Hospital, Jackson, MI 49201, USA; pgrover1@hfhs.org; 2 Department of Internal Medicine, Sri Manakula Vinayagar Medical College, Puducherry 605107, India; rahul.jainmanohar@gmail.com; 3 Department of Internal Medicine, Government Medical College, Amritsar 143001, India; drgurleenkaur25@gmail.com; 4 Department of Gastroenterology, University of Kentucky, Lexington, KY 40506, USA

**Keywords:** interstitial lung disease, idiopathic pulmonary fibrosis, systemic sclerosis

## Abstract

**Highlights:**

**What are the main findings?**

- Males accounted for 53–55% of ILD deaths, with the proportion rising from 51.2% (1999) to 54.8% (2024).
- White–Black AAMR ratio widened from 1.48:1 (1999) to 1.79:1 (2020)

**What are the implications of the main findings?**

- Deaths attributed to selected J84-coded ILDs increased substantially, while age-adjusted mortality increased more slowly.
- No temporal reduction in population mortality coincided with the 2014 introduction of antifibrotic therapy.

**Abstract:**

Interstitial lung diseases (ILDs) comprise a heterogeneous group of pulmonary disorders associated with substantial morbidity and mortality. We examined mortality attributed to selected J84-coded ILDs in the United States from 1999 to 2024 using CDC WONDER underlying-cause-of-death data. Age-adjusted mortality rates (AAMRs) per 100,000 population were standardized to the 2000 U.S. population and stratified by sex and race. Joinpoint regression was used to identify changes in temporal slope and estimate annual percent change (APC), average annual percent change (AAPC), 95% confidence intervals (CIs), and *p*-values. After the removal of overlapping years between the CDC WONDER database series, 444,573 unique deaths occurred. Annual deaths increased from 11,358 in 1999 to 22,849 in 2024, while AAMR increased from 4.2 to 5.1 per 100,000. Overall, AAMR increased during 1999–2004 (APC 2.32%, 95% CI 1.39–3.26; *p* < 0.001) and more slowly during 2004–2024 (APC 0.36%, 95% CI 0.16–0.56; *p* = 0.001), with an overall AAPC of 0.75% (95% CI 0.60–0.90; *p* < 0.001). Male AAMRs remained higher than female AAMRs, while race-specific trends were heterogeneous. No temporal reduction in population mortality coincided with the introduction of antifibrotic therapies; however, this ecological analysis cannot evaluate treatment effectiveness or individual treatment exposure.

## 1. Introduction

Reported ILD frequency varies substantially by case definition, population, and ascertainment method. In the United States and internationally, aging populations and improved case ascertainment may contribute to observed increases in diagnosed disease; however, mortality data alone cannot determine changes in incidence, prevalence, or overall disease burden.

Sarcoidosis, connective tissue disease-associated ILD (CTD-ILD), and idiopathic pulmonary fibrosis (IPF) are the three most common fibrotic ILDs, with estimated prevalences of 30.2, 12.1, and 8.2 per 100,000, respectively [1,2,3,4]. Idiopathic pulmonary fibrosis occurs in people aged over 60 years, and it affects men more than women. In the United States, IPF prevalence ranges from 14 to 63 per 100,000 and was as high as 494 per 100,000 among adults over 65 in one study [3]. Males consistently have higher incidence and mortality rates than females across all regions [3]. Globally, ILD-related deaths totaled 169,833 in 2019, with a 166.6% increase in deaths from 1990 to 2019 [4]. Connective tissue-induced ILD occurs in individuals aged 40–60 years [5]. It is more common in females than in males. CTD-ILD prevalence varies by underlying disease: 65% of patients with systemic sclerosis and up to 80% of those with diffuse cutaneous systemic sclerosis develop ILD, while 36–45% of patients with idiopathic inflammatory myopathy develop ILD (up to 80% in those with specific antisynthetase antibodies) [5]. Incidence estimates for individual non-IPF subtypes are lower: Idiopathic NSIP 0.8 per 100,000/year, CTD-ILD ~2.7–4.3 per 100,000/year, fibrotic hypersensitivity pneumonitis 1.1 per 100,000/year, and sarcoidosis with ILD 4.9 per 100,000/year [3].

Nintedanib and pirfenidone slow disease progression in idiopathic pulmonary fibrosis. Because the present study evaluates mortality trends surrounding the 2014 approval of these agents, newer therapies and investigational agents outside this time frame are not used to interpret the pre- versus post-2014 comparison.

We aimed to (1) characterize temporal trends in mortality attributed to selected J84-coded ILDs using Joinpoint regression, (2) quantify sex- and race-specific differences with uncertainty estimates, and (3) describe whether any temporal change in population mortality coincided with the 2014 introduction of antifibrotic therapy, without inferring individual treatment effectiveness.

## 2. Materials and Methods

### 2.1. Study Design and Data Source

This was a retrospective, population-based cross-sectional study of mortality trends and demographic disparities in ILD in the United States from 1999 to 2024. Mortality data were obtained from the Centers for Disease Control and Prevention’s Wide-ranging Online Data for Epidemiologic Research (CDC WONDER) Underlying Cause of Death databases. 

Two databases were queried: the 1999–2020 series (bridged-race population estimates) and the 2018–2024 Single Race series (single-race population estimates). 

Deaths were identified when ICD-10 codes J84.0, J84.1, J84.8, or J84.9 were listed as the underlying cause of death. Accordingly, this study does not represent all ILD mortality. Important ILD-related conditions coded outside J84, including sarcoidosis (D86), hypersensitivity pneumonitis (J67), and some connective tissue disease-associated ILDs, were not captured, while the included J84 codes combine clinically heterogeneous disorders. We therefore describe the outcome as mortality attributed to selected J84-coded ILDs rather than all ILD or total ILD disease burden.

For the merged 1999–2024 dataset, data from 1999 to 2017 were drawn from the 1999–2020 series, and data from 2018 to 2024 were drawn from the 2018–2024 Single Race series. The 1999–2020 series uses bridged-race population estimates, while the 2018–2024 series uses single-race population estimates; this methodological difference is noted as a limitation for race-stratified analyses spanning both databases.

### 2.2. Outcome Measures

The primary outcome was the age-adjusted mortality rate (AAMR) per 100,000 population, standardized to the 2000 U.S. standard population using the direct method. 

Secondary outcomes included absolute annual death counts, crude mortality rates per 100,000 population, and mortality rate ratios with 95% confidence intervals for demographic comparisons.

### 2.3. Demographic Variables

Deaths were stratified by the following variables as recorded on the death certificate:

- Sex: Female and male.
- Race: For the 1999–2020 series, race was categorized as White, Black or African American, American Indian or Alaska Native, and Asian or Pacific Islander. For the 2018–2024 series, the Single Race 6 classification was used: White, Black or African American, Asian, Native Hawaiian or Other Pacific Islander, American Indian or Alaska Native, and More than one race.

Four pre-specified analyses were performed. Annual AAMRs were evaluated using segmented log-linear Joinpoint regression. Candidate breakpoints were assessed across the annual series with a minimum of three observed years per segment, and the best-fitting model was selected using the Bayesian information criterion. APC, AAPC, 95% CIs, and *p*-values were estimated. Separate models were fitted for the overall population and for each sex and racial group. CDC WONDER gamma-method 95% CIs were retained for annual AAMRs. Because this ecological analysis contains no individual treatment information, the 2014 antifibrotic approval date was used only as a temporal context and not as a causal intervention point.

## 3. Results

From 1999 to 2024, 444,573 unique deaths were attributed to selected J84-coded ILDs as the underlying cause of death in the United States. The previously reported total of 505,816 incorrectly added both the CDC WONDER series and double-counted 61,243 deaths during the overlapping years 2018–2020. The corrected merged dataset uses 1999–2017 from the 1999–2020 series and 2018–2024 from the Single Race series. Annual deaths increased from 11,358 in 1999 to 22,849 in 2024, a 101% increase in absolute counts, while the crude mortality rate rose from 4.1 to 6.7 per 100,000. Table 1 denotes the year-wise death, crude rate, and AAMR reporting with year-over-year death change.

**Figure 1:**
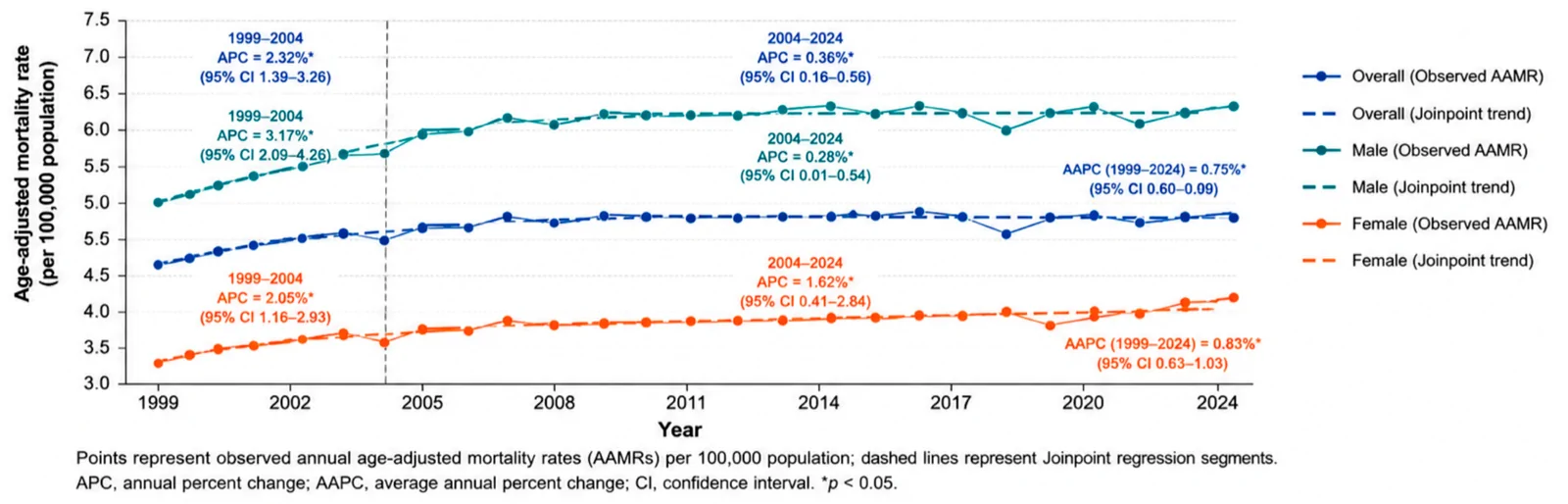
Annual age-adjusted mortality rates and fitted Joinpoint segments overall and by sex.

The male–female AAMR ratio (Table 2) remained persistently elevated. Female AAMR increased during 1999–2004 (APC 3.17%, 95% CI 2.09–4.26; *p* < 0.001), was stable during 2004–2020 (APC −0.08%, 95% CI −0.41 to 0.24; *p* = 0.594), and increased during 2020–2024 (APC 1.62%, 95% CI 0.41–2.84; *p* = 0.011). Male AAMR increased during 1999–2006 (APC 2.05%, 95% CI 1.16–2.93; *p* < 0.001) and more slowly during 2006–2024 (APC 0.28%, 95% CI 0.01–0.54; *p* = 0.041). Overall AAPCs were 0.83% (95% CI 0.63–1.03) for females and 0.77% (95% CI 0.57–0.97) for males. These population-level rate differences should not be interpreted as individual susceptibility or risk. Figure 1 shows formal joinpoint analysis by sex.

**Figure 2:**
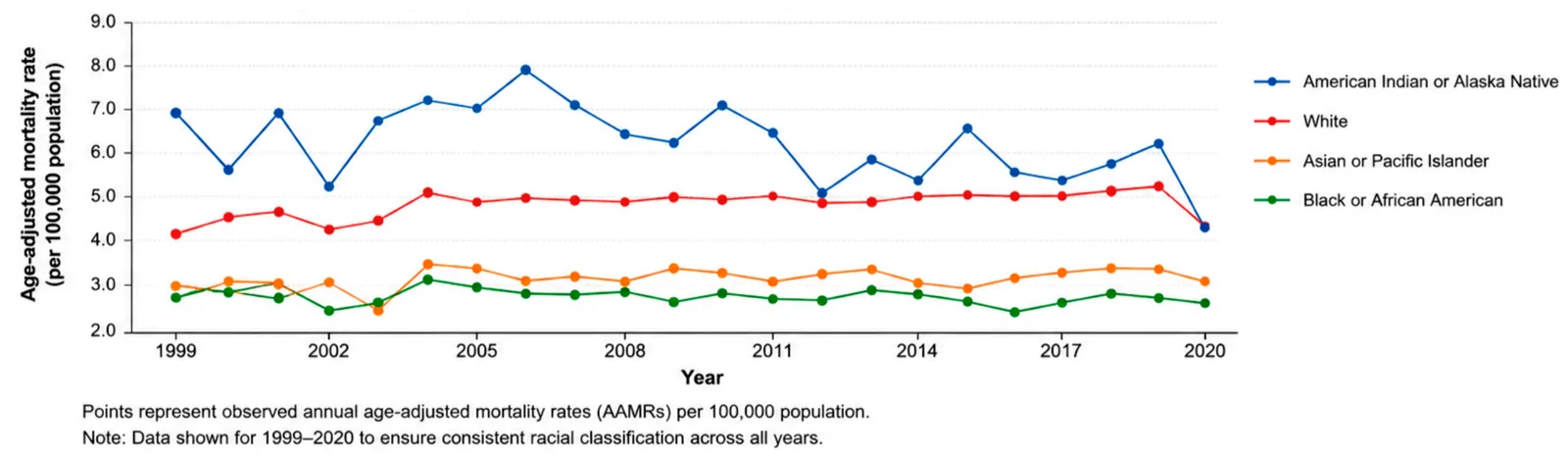
Annual age-adjusted mortality rates by race.

Race-specific Joinpoint models showed heterogeneous trends (Table 3, Figure 2). White AAMR increased during 1999–2006 (APC 2.36%, 95% CI 1.62–3.10; *p* < 0.001) and more slowly during 2006–2020 (APC 0.46%, 95% CI 0.15–0.76; *p* = 0.005), yielding an AAPC of 1.09% (95% CI 0.89–1.28). Full-period trends for Black or African American individuals (AAPC −0.35%, 95% CI −0.74 to 0.05) and Asian or Pacific Islander individuals (AAPC 0.48%, 95% CI −0.14 to 1.10) were not statistically significant. American Indian or Alaska Native rates were variable and showed an overall decrease (AAPC −0.96%, 95% CI −1.90 to −0.01), but these estimates warrant caution because of smaller counts and known race misclassification. The White–Black AAMR ratio increased descriptively from 1.48 in 1999 to 1.79 in 2020; this should not be interpreted as evidence of individual-level racial susceptibility.

The pre-specified 2014 approval date did not correspond to an identified Joinpoint breakpoint; the overall model identified a breakpoint in 2004. Thus, no temporal reduction in population mortality coincided with the introduction of antifibrotic therapies. This finding cannot estimate treatment effectiveness because the dataset lacks ILD subtype, treatment eligibility, medication exposure, uptake, adherence, disease severity, referral patterns, and transplantation data, and because antifibrotics were initially approved for IPF rather than the broader set of J84-coded conditions analyzed here. Table 4 compares the outcomes before and after 2014.

## 4. Discussion

This population-based analysis of CDC WONDER data spanning 26 years identified 444,573 unique deaths attributed to selected J84-coded ILDs. Absolute annual deaths increased substantially, and Joinpoint regression showed a significant increase in AAMR, with faster growth during 1999–2004 and slower growth thereafter. Mortality is only one component of disease burden; therefore, these findings should not be interpreted as estimates of ILD prevalence, disability, hospitalization, healthcare utilization, or quality of life.

As the population aged 65 years and older expanded from approximately 35 million in 1999 to over 60 million by 2024, the absolute number of individuals at risk for ILD-related death increased substantially, even without a proportional increase in age-specific risk. This finding aligns with Spagnolo et al. (2025), who noted that ILD mortality rates are generally higher in people older than 55 years and that the burden on the elderly population is garnering growing attention as the global population ages [6]. Similar observations have also been reported in a recent Global Burden of Disease analysis of adults aged ≥55 years, which demonstrated increasing mortality and disability attributable to ILD and pulmonary sarcoidosis in older populations [7].

The 2020 decrease and subsequent rebound occurred during the COVID-19 pandemic. Possible explanations include changes in underlying-cause coding, disruption of healthcare access, competing attribution of deaths to COVID-19, delayed care, or post-COVID pulmonary sequelae. These mechanisms were not measured in the present dataset and remain hypotheses rather than demonstrated explanations. Patients with pre-existing ILD experienced substantially worse outcomes following COVID-19 infection in international cohort studies and meta-analyses, providing biological plausibility that the pandemic may have influenced mortality patterns during this period [8,9]. A recent U.S. mortality analysis similarly reported temporal changes in ILD mortality during the COVID-19 era [10].

The persistently higher male AAMR is consistent with prior epidemiologic observations in ILD and IPF. Potential contributors described in other studies include differences in ILD subtype distribution, age, smoking, and occupational or environmental exposures. Recent registry data have also demonstrated sex-specific phenotypes and clinical outcomes among patients with non-IPF ILD [11]. However, these variables were not available in CDC WONDER and therefore cannot explain the sex difference observed here. The present results establish a population-level rate difference, not greater individual susceptibility among males.

Race-specific mortality patterns require cautious interpretation. White individuals had the highest AAMR in this dataset, and the White–Black AAMR ratio increased descriptively over time. These findings do not establish biological susceptibility or individual-level risk and may reflect differences in disease subtype distribution, diagnosis, access to specialty care, competing causes of death, coding practices, and population denominators. Interpretation is further limited by death-certificate race misclassification, the transition from bridged-race to single-race population estimates, lack of ethnicity-specific analysis, and statistical instability in smaller groups.

The temporal comparison around 2014 should not be interpreted as an evaluation of antifibrotic effectiveness. The Joinpoint model did not identify 2014 as a breakpoint, and no temporal decline in population mortality coincided with antifibrotic introduction. Moreover, antifibrotics were initially approved for IPF, whereas this study includes a broader set of selected J84-coded ILD deaths. Individual-level studies and meta-analyses have demonstrated mortality benefits of antifibrotic therapy in patients with IPF [12,13,14,15], and more recently, newer therapeutic agents including nerandomilast and pamrevlumab have shown promise in clinical trials [16,17]. However, these studies evaluate treatment efficacy under specific clinical conditions and therefore address a fundamentally different causal question from the ecological mortality trends examined here.

This study has several limitations. First, underlying-cause-of-death data capture only deaths for which a selected J84 code was designated as initiating the fatal sequence and do not measure the full clinical or societal burden of ILD. Mortality does not capture prevalence, disability, hospitalization, healthcare utilization, or quality of life. Second, ICD coding accuracy could not be independently validated, incident disease could not be identified, and the selected J84 codes both exclude important ILD-related conditions and combine heterogeneous entities. A future multiple-cause-of-death sensitivity analysis could capture deaths in which ILD contributed but was not selected as the underlying cause. Third, CDC WONDER contains no individual-level information on ILD subtype, disease severity, comorbidities, referral, transplantation, or antifibrotic exposure, uptake, adherence, or adverse effects; causal inference regarding treatment is therefore not possible. Fourth, race may be misclassified on death certificates, particularly for American Indian/Alaska Native and multiracial individuals; estimates also span a transition from bridged-race to single-race population denominators, ethnicity-specific trends were not examined, and smaller groups had unstable annual rates. Finally, Joinpoint analysis identifies statistical changes in temporal slope but cannot determine the mechanisms responsible for those changes.

## 5. Conclusions

Deaths attributed to selected J84-coded ILDs increased substantially in the United States from 1999 to 2024. Joinpoint regression showed a significant increase in AAMR, with faster growth during 1999–2004 and slower growth thereafter. Male AAMRs remained higher than female AAMRs, and race-specific trends were heterogeneous, but these ecological differences should not be interpreted as individual susceptibility or causation. The 2014 introduction of antifibrotic therapy did not coincide with a population-level decline in mortality; however, the absence of such a temporal decline does not indicate treatment failure because treatment exposure and multiple clinical determinants of mortality were unavailable. These findings describe long-term mortality patterns and support continued surveillance.

## Figures and Tables

**Table 1:** The 1999–2024 year-wise death, crude rate, AAMR, and year-over-year death change.

Year	Deaths	Crude Rate	AAMR	Year-Over-Year Death Change
1999	11,358	4.1	4.2	—
2000	12,374	4.4	4.5	+8.9%
2001	13,068	4.6	4.7	+5.6%
2002	12,017	4.2	4.2	−8.0%
2003	12,699	4.4	4.4	+5.7%
2004	14,975	5.1	5.1	+17.9%
2005	14,376	4.9	4.8	−4.0%
2006	14,834	5.0	4.9	+3.2%
2007	15,084	5.0	4.9	+1.7%
2008	15,665	5.2	5.0	+3.9%
2009	15,930	5.2	4.9	+1.7%
2010	16,261	5.3	5.0	+2.1%
2011	16,947	5.4	5.1	+4.2%
2012	17,005	5.4	5.0	+0.3%
2013	17,586	5.6	5.0	+3.4%
2014	18,023	5.7	5.0	+2.5%
2015	18,488	5.8	5.0	+2.6%
2016	18,722	5.8	5.0	+1.3%
2017	19,615	6.0	5.1	+4.8%
2018	20,492	6.3	5.2	+4.5%
2019	21,221	6.5	5.2	+3.6%
2020	19,530	5.9	4.7	−8.0%
2021	21,178	6.4	5.3	+8.4%
2022	21,609	6.5	5.1	+2.0%
2023	22,667	6.8	5.3	+4.9%
2024	22,849	6.7	5.1	+0.8%

**Table 2:** AAMR distribution based on patient sex.

Year	Female AAMR	Male AAMR	Male–Female Ratio
1999	3.4	5.4	1.59
2000	3.6	5.9	1.64
2001	3.9	5.8	1.49
2002	3.5	5.4	1.54
2003	3.6	5.6	1.56
2004	4.2	6.5	1.55
2005	3.9	6.1	1.56
2006	3.9	6.3	1.62
2007	3.9	6.3	1.62
2008	4.0	6.4	1.60
2009	4.0	6.4	1.60
2010	4.0	6.5	1.63
2011	4.1	6.5	1.59
2012	4.0	6.3	1.58
2013	3.9	6.4	1.64
2014	3.9	6.5	1.67
2015	3.9	6.5	1.67
2016	3.9	6.4	1.64
2017	4.0	6.5	1.63
2018	4.1	6.6	1.61
2019	4.1	6.8	1.66
2020	3.7	6.1	1.65
2021	4.1	6.9	1.68
2022	4.0	6.6	1.65
2023	4.2	6.7	1.60
2024	4.2	6.5	1.55

**Table 3:** Age-adjusted mortality rates (AAMRs) by race from 1999 to 2020.

Year	White AAMR	Black AAMR	Asian/PI AAMR	AI/AN AAMR	White–Black Ratio
1999	4.3	2.9	3.1	7.4	1.48
2000	4.6	3.2	3.0	5.7	1.44
2001	4.8	3.2	2.8	7.2	1.50
2002	4.4	2.6	3.2	5.2	1.69
2003	4.6	2.7	2.5	6.9	1.70
2004	5.3	3.3	3.6	7.3	1.61
2005	5.0	3.1	3.6	7.1	1.61
2006	5.1	3.0	3.2	7.9	1.70
2007	5.1	3.0	3.3	7.2	1.70
2008	5.2	3.1	3.2	6.6	1.68
2009	5.2	2.8	3.5	6.4	1.86
2010	5.2	3.0	3.4	7.2	1.73
2011	5.3	2.9	3.2	6.5	1.83
2012	5.2	2.9	3.4	4.8	1.79
2013	5.2	3.1	3.5	6.0	1.68
2014	5.3	3.0	3.1	5.4	1.77
2015	5.3	2.8	3.1	6.7	1.89
2016	5.3	2.6	3.4	5.6	2.04
2017	5.4	2.8	3.5	5.4	1.93
2018	5.5	3.0	3.6	5.8	1.83
2019	5.5	2.9	3.6	6.4	1.90
2020	5.0	2.8	3.1	4.3	1.79

**Table 4:** Mortality before (1999–2014) and after (2015–2024) the introduction of antifibrotic therapies.

Parameter	Pre-Antifibrotic (1999–2014)	Post-Antifibrotic (2015–2024)
Duration (years)	16	10
Total deaths	238,202	206,371
Mean annual deaths	14,888	20,637
Crude rate range (per 100,000)	4.1–5.7	5.8–6.8
Aggregate AAMR (per 100,000)	4.8	5.1
AAMR range (per 100,000)	4.2–5.1	4.7–5.3

## Data Availability

The original contributions presented in this study are included in the article. Further inquiries can be directed to the corresponding author.
